# Compact octagonal MIMO antenna system for broadband applications with enhanced isolation and wideband performance

**DOI:** 10.1038/s41598-025-03494-7

**Published:** 2025-05-29

**Authors:** R. Dhananjeyan, S. Ramesh, D. Rajesh Kumar, Om Prakash Kumar

**Affiliations:** 1 Department of Electronics and Communication Engineering, SRM Valliammai Engineering College, Chennai, India; 2 Department of Electronics and Communication Engineering, Vel Tech Rangarajan Dr. Sagunthala R&D Institute of Science and Technology, Chennai, Tamilnadu India; 3 Department of Electronics and Communication Engineering, Manipal Institute of Technology, Manipal Academy of Higher Education, Manipal, 576104 India

**Keywords:** MIMO, Current limiting stub, ECC, CCL, DG, Sdg 9: industry, innovation, and infrastructure, SDG 11: sustainable cities and communities

## Abstract

This work introduces a compact and novel MIMO antenna system with close spacing and exceptional isolation, tailored for broadband usage across frequencies from 3.7 to 11 GHz. The antenna configuration consists of two octagonal radiators, each individually powered by a 50 Ω microstrip line. The use of a modified ground structure ensures a wideband frequency range. A key novelty of this design is the incorporation of a strategically placed stub (25.6875 × 0.5 mm^2^) between the radiating sections, which significantly enhances inter-port isolation, achieving over 15 dB despite the minimal 3 mm port separation—an improvement over conventional MIMO antennas. This design enables high isolation without increasing antenna size, making it highly efficient for compact wireless systems. Performance evaluation, including fabrication and testing, demonstrates a strong alignment between simulation and experimental data. Metrics such as envelope correlation coefficient (ECC), diversity gain (DG), and channel capacity loss (CCL) were analyzed to assess MIMO efficiency. The proposed antenna is ideal for broadband wireless systems with a peak gain of 6.5 dBi and excellent resonance, offering superior isolation and compactness compared to existing designs.

## Introduction

The development of multiple-input multiple-output (MIMO) antenna systems has become pivotal in addressing the ever-growing demand for high data rates and reliable communication in wireless systems. Researchers have explored various design approaches to optimize performance metrics such as isolation, bandwidth, gain, and compactness, particularly for ultra-wideband (UWB) and 5G applications. Several researchers have focused on UWB-MIMO antennas to achieve wide bandwidth and improved isolation. Haripriya et al.^1^ introduced a high-isolation two-element UWB-MIMO antenna with a WLAN single-band notched feature, utilizing Roger material for enhanced performance. Similarly, Hiwa et al.^2^ presented a novel antenna design tailored for microwave communications, emphasizing compactness and high isolation. Other works, such as those by Jayshri et al.^3^, proposed a four-port MIMO antenna array with high isolation, suitable for next-generation wireless systems.The integration of advanced materials and structures has significantly contributed to the optimization of MIMO antenna systems. Kulkarni et al.^4^ demonstrated the use of flexible interconnected four-port MIMO antennas for sub-6 GHz 5G and X-band applications, highlighting adaptability for diverse environments. Ekrami and Jam^5^ explored compact triple-band dual-element antennas, achieving high port-to-port isolation through novel structural arrangements. Meanwhile, Alibakhshikenari et al.^6^ utilized metasurface inclusions to reduce surface wave effects, enhancing isolation and performance in MIMO and SAR systems.Metamaterials and defected ground structures (DGS) have been extensively employed to achieve band rejection and mutual coupling reduction. Vishal Puri and Hari Shankar Singh^7^ utilized a meta-surface-based absorber to enhance isolation in MIMO antennas, while Hassan et al.^8^ integrated a U-shaped stub-loaded DGS in their monopole UWB-MIMO design. Similarly, Shuang et al.^9^ presented a dual-port UWB-MIMO antenna with quadruple band-notched characteristics, addressing interference issues effectively.With the increasing focus on compactness, several researchers have proposed designs suitable for wearable and flexible applications. Abdalla and Ibrahim^10^ introduced metamaterial-inspired MIMO antennas with compact dimensions and high efficiency for wireless systems. Likewise, Iqbal et al.^11^ developed low-profile, closely spaced four-element MIMO antennas for wireless body area networks, emphasizing comfort and practicality.Isolation remains a critical factor in MIMO antenna design. Techniques such as the use of parasitic elements, decoupling networks, and neutralization lines have been widely studied. Phuong et al.^12^ utilized parasitic elements for simultaneous improvements in isolation, bandwidth, and gain, while Jiang et al.^13^ employed combined decoupling networks in their 5G MIMO antenna design. Furthermore, Kayabasi et al.^14^ enhanced isolation using a neutralization ring in their triangular quad-port UWB-MIMO antenna.Application-specific requirements have also driven antenna innovations. For instance, Tao and Feng^15^ designed a compact UWB-MIMO antenna with a half-slot structure tailored for vehicular communications, addressing the unique challenges posed by such environments. Similarly, Prabhu and Malarvizhi^16^ proposed a polarization diversity 3D-UWB MIMO antenna with dual-band notch characteristics, catering to vehicular communication needs.The continuous advancements in MIMO antenna technology underscore the importance of innovative design methodologies to meet the demands of modern wireless communication systems. From UWB-MIMO designs with enhanced isolation to compact, application-specific solutions, these contributions pave the way for more efficient and reliable communication systems in the future.

In this study, we have presented an octagonal shaped two-element antenna for use in wide-band applications spanning between 3.7 and 11 GHz. The two antennas are positioned with a separation of 3 mm and a long rectangular stub is placed between them to restrict current flow between the antenna elements. The end result is a substantial enhancement in isolation, above 15 dB across the whole operational bandwidth, thereby obtaining considerable MIMO characteristics Based on the results of this investigation, it appears that the antenna that was suggested for use in broad-band applications would be a good option for such applications.

The selection of an octagonal radiator shape over conventional geometries is driven by its ability to enhance bandwidth, isolation, and radiation efficiency. Unlike rectangular or circular designs, the octagonal shape provides a larger effective radiating surface and supports multiple resonant modes, leading to improved impedance bandwidth. Its symmetrical structure ensures a more uniform current distribution, reducing losses and enhancing gain. Additionally, the octagonal geometry allows better integration of decoupling techniques, such as electromagnetic bandgap structures or neutralization lines, which improve isolation in MIMO applications. This design choice balances compactness with superior electromagnetic performance, making it ideal for broadband MIMO antenna systems.

To make readers comfort structure of the article is decomposed into four sections. Section I briefs MIMO antenna structure and analysis. Evolutionary stages of proposed antenna displayed under Section II followed by effect of current limiting stub labelled under Section III. Section IV focuses on measured results and discussion whereas Section V concentrates on various MIMO characteristics.

### The following could potentially be applied to describe the envisioned work’s significance

1. The MIMO antenna presented here occupies a minimal area of approximately 38 Χ 25 mm^2^, which is a significantly smaller than previous works published on the subject.
2. Using this type of low-profile configuration, the antenna is capable of broad-band operation, the frequency range of interest is from 3.7 to 11 GHz, and it has an impedance bandwidth of 10 dB.
3. The antenna system that was implemented consists of two elements MIMO that achieves over 15 dB of isolation by integrating a current limiting stub between the elements.
4. The most important characteristic of the antenna is that it can attain a maximal gain of 6.5 dBi, which is sufficient for broad-band wireless applications.
5. Further antenna possesses substantial MIMO capabilities that cover the entire operational spectrum and include ECC, DG, and CCL.

## MIMO antenna design and analysis

**Fig. 1:**
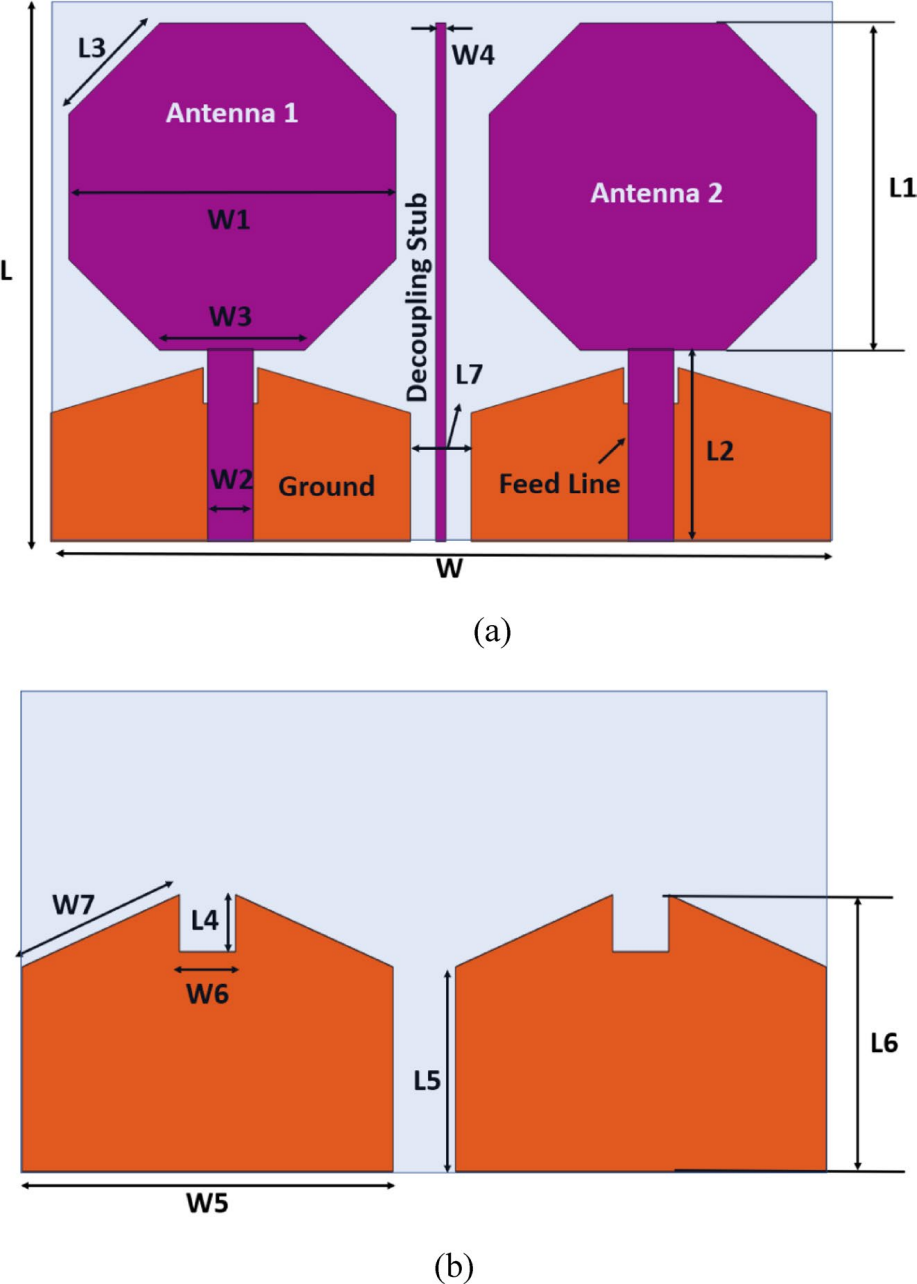
The proposed single element antenna’s structure and dimensions. (**a**) Front View, (**b**) back view.

Figure 1a,b illustrate the structure and dimensions of the proposed single-element antenna, showing the front view of the dual-antenna configuration and providing a detailed view of the ground plane design respectively. In Fig. 1a, two octagonal radiating elements, labeled Antenna 1 and Antenna 2, are separated by a decoupling stub to reduce mutual coupling and interference. The layout includes labeled dimensions for the feed line (W2, L2) and the ground plane, all encapsulated within a rectangular area defined by W (width) and L (length). Figure 1b highlights the slotted ground plane, featuring triangular and rectangular cutouts defined by dimensions such as L4, W4, W6, and W7, designed to enhance antenna performance by improving impedance matching and radiation characteristics. This compact and optimized structure is well-suited for applications like multiple-input multiple-output (MIMO) systems.A detailed summary of the individual component dimensions is provided in Table 1, ensuring reproducibility and clarity for the proposed design. The novelty of the proposed MIMO antenna lies in its compact design, exceptional isolation, and broadband capability. Unlike conventional MIMO antennas, this design achieves high isolation (over 15 dB) despite a minimal 3 mm element spacing, which is made possible by a strategically placed 25.6875 × 0.5 mm^2^ stub and a modified ground structure. The antenna operates over an ultra-wideband frequency range (3.7–11 GHz), ensuring versatile applicability in broadband wireless systems. Additionally, the strong correlation between simulation and experimental results validates its real-world performance, making it a highly efficient and innovative solution for modern MIMO communication systems.

**Table 1:** Various dimensions of an individual antenna element.

Parameters	L	L1	L2	L3	L4	L5	L6	L7	W	W1	W2	W3	W4	W5	W6	W7
Values (mm)	27	16	9.5	4.5	1.77	6.36	8.6	3	38.64	16.1	2.25	7.2	0.5	17.82	2.7	7.56

**Fig. 2:**
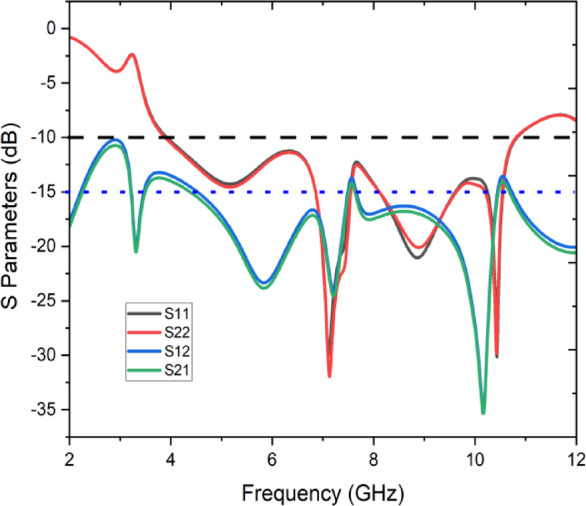
S-Parameters of the Proposed two-element antenna system.

Figure 2 illustrates the S-parameters of the proposed two-element antenna system, representing key performance metrics over a frequency range of 2 GHz to 12 GHz. The parameters S11and S22 correspond to the reflection coefficients of Antenna 1 and Antenna 2, respectively, indicating how well the antennas match the feed line. Both S11 and S22 exhibit values below − 10 dB across multiple frequency bands, signifying effective impedance matching and good return loss performance. The parameters S12 and S21 represent the transmission coefficients between the two antennas, which measure the level of mutual coupling. These values remain significantly below − 15 dB across the operational bands, demonstrating strong isolation between the antenna elements. This high isolation ensures minimal interference, which is critical for applications such as MIMO systems, where independent operation of antenna elements is essential. The overall performance indicates a well-designed system optimized for wideband operation and low mutual coupling.

## Design process

**Fig. 3:**
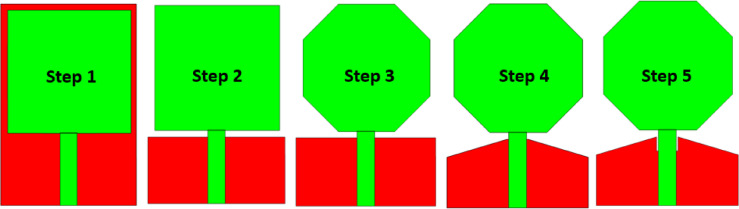
Phases of design for the proposed antenna.

**Fig. 4:**
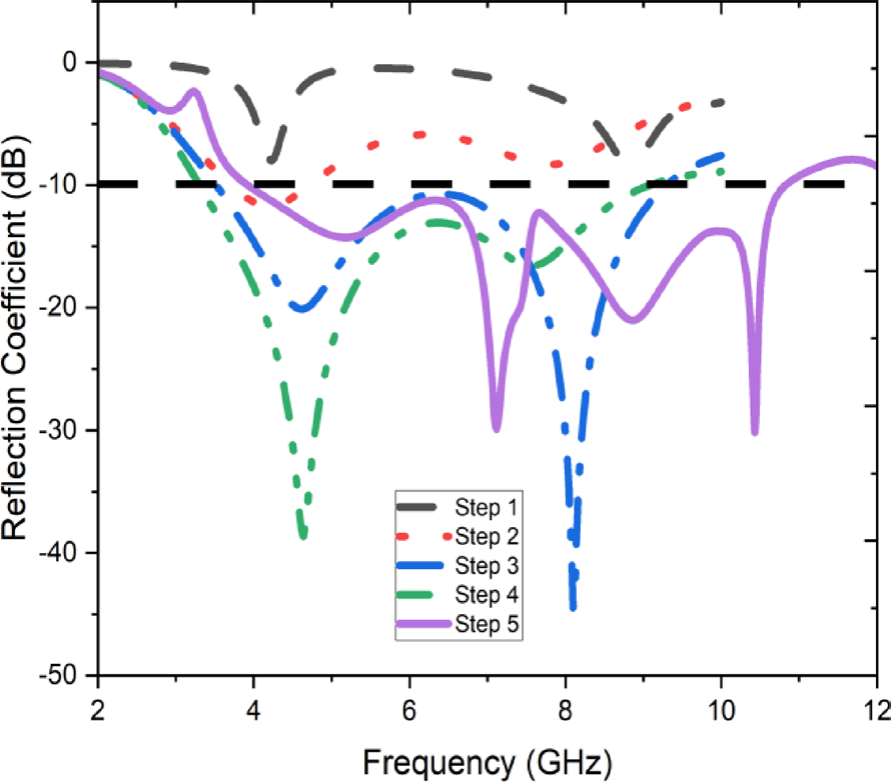
Review of the proposed antenna’s performance during the design process.

Figure 3 demonstrates the step-by-step design evolution of the proposed antenna through five phases. In Step 1, the design begins with a simple rectangular radiating patch connected to a feed line, supported by a full rectangular ground plane. In Step 2, the ground plane is partially modified with cutouts to improve impedance matching and enhance the bandwidth. Step 3 transitions the radiating element to an octagonal shape, which optimizes current distribution and minimizes edge diffraction, enhancing the radiation characteristics. In Step 4, triangular cutouts are introduced in the ground plane to further optimize impedance and reduce mutual coupling. Finally, Step 5 incorporates additional refinements to both the radiating element and the ground plane, resulting in a compact structure with improved bandwidth, return loss, and isolation, tailored for high-performance applications like MIMO systems. Figure 4 illustrates the reflection coefficient S11 performance of the proposed antenna across different design stages (Steps 1 to 5). Each step represents an iterative modification in the design process, aiming to optimize impedance matching and broaden the operational bandwidth. In Step 1 (red dashed line), the initial design shows a limited response with poor resonance characteristics. Incremental adjustments in subsequent steps (Steps 2 to 4) result in progressive improvements, particularly around the targeted frequency ranges, as indicated by deeper nulls in the reflection coefficient. The final design (Step 5, purple line) achieves multiple resonances, notably below \(-10\ \text{dB}\) at several frequency bands, demonstrating significant enhancement in impedance matching. This step effectively covers the intended frequency ranges, confirming the antenna’s suitability for its designated application. The figure also highlights the optimization process, showcasing how specific adjustments influence the antenna’s performance across the spectrum.

## Isolation Enhancement approach: Current limiting stub

The stub is an effective technique for improving inter-port isolation in MIMO antennas by acting as a decoupling structure that suppresses mutual coupling between antenna elements. It functions by introducing a high-impedance path or counteracting coupled signals, thereby reducing unwanted interference. Compared to other isolation techniques, such as electromagnetic bandgap (EBG) structures, defected ground structures (DGS), or neutralization lines, the stub offers a compact and low-complexity solution while maintaining wideband performance. Unlike EBG and DGS, which may require complex fabrication and tuning, or neutralization lines that need precise design adjustments, stubs provide a simple yet effective means of enhancing isolation with minimal impact on gain and bandwidth. When optimized, stub-based isolation techniques can achieve inter-port isolation levels exceeding − 20 dB, making them highly suitable for broadband MIMO applications.

**Fig. 5:**
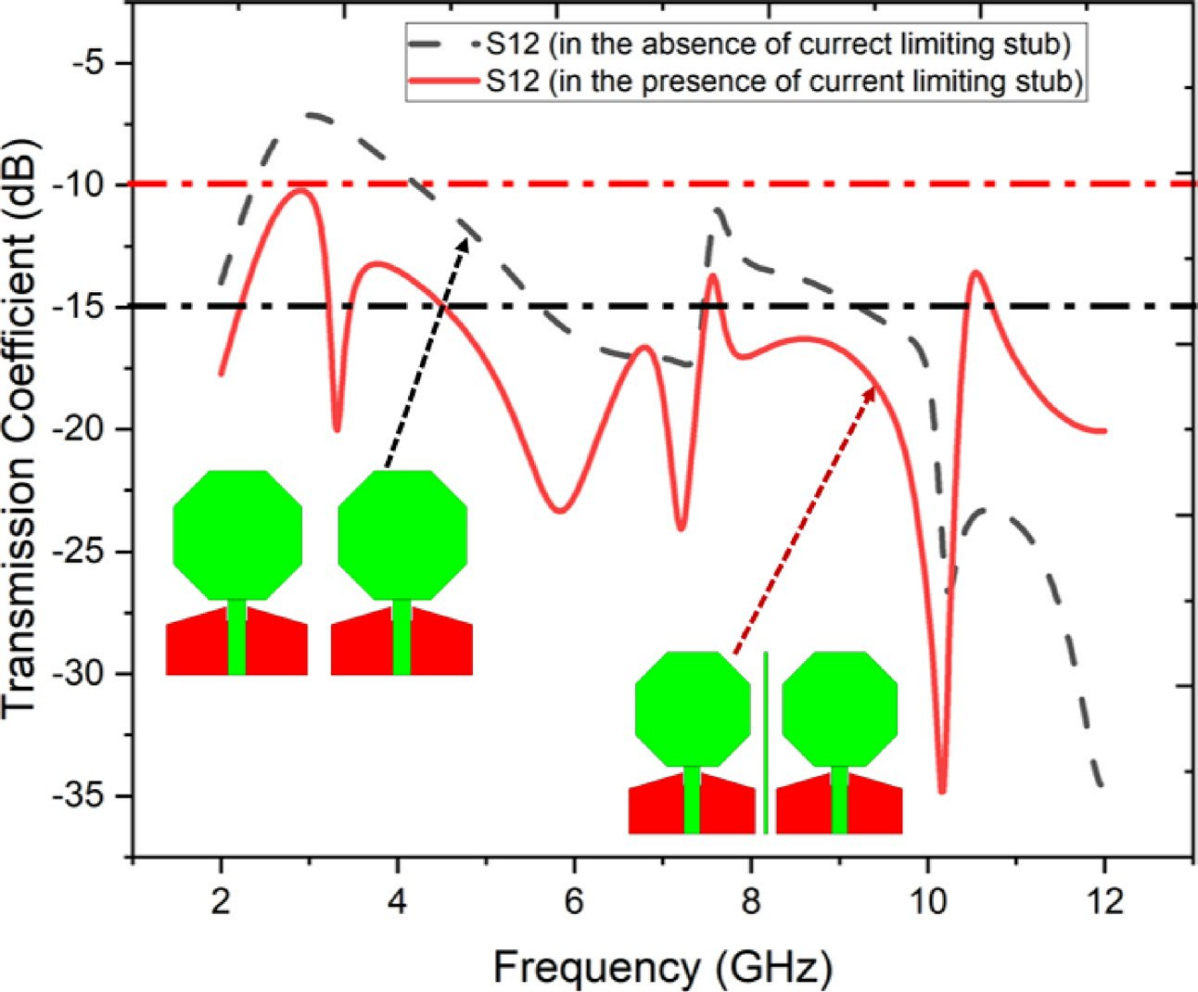
Performance analysis of the proposed antenna with and without decoupling stub.

In MIMO antenna design, achieving effective isolation between radiating components is crucial for improving overall radiation performance. This section outlines the isolation enhancement techniques employed in this research. Despite the minimal separation of just 3 mm between the two antennas in the proposed MIMO system, an isolation of more than 15 dB across the entire operating band is achieved by incorporating a current-limiting stub between them. Figure 5 compares the transmission coefficients of the MIMO antenna system with and without the decoupling stub. Without the stub, the transmission coefficient ranges from 11 dB to 16.5 dB. However, with the inclusion of the current-limiting stub, the maximum transmission coefficient improves significantly, reaching 24 dB at 7 GHz. This demonstrates the effectiveness of the decoupling stub in enhancing isolation, even with the antennas separated by a minimal distance of 3 mm. Figure 5 demonstrates the impact of incorporating a current-limiting stub on the isolation S12 performance of the proposed antenna. The dashed black line represents the transmission coefficient S12 without the stub, while the solid red line corresponds to the scenario with the stub included. Without the stub, the antenna exhibits poor isolation, with S12 values closer to – 10 dB across certain frequency ranges, indicating significant mutual coupling between ports. The addition of the current-limiting stub significantly reduces coupling, achieving isolation levels below − 20 dB across the majority of the operating spectrum. This improvement is particularly evident around the target frequency bands, where the stub effectively suppresses unwanted current pathways. The visual insets further highlight the structural differences in the antenna design with and without the stub, underscoring its critical role in enhancing isolation for MIMO or multi-port applications.

**Fig. 6:**
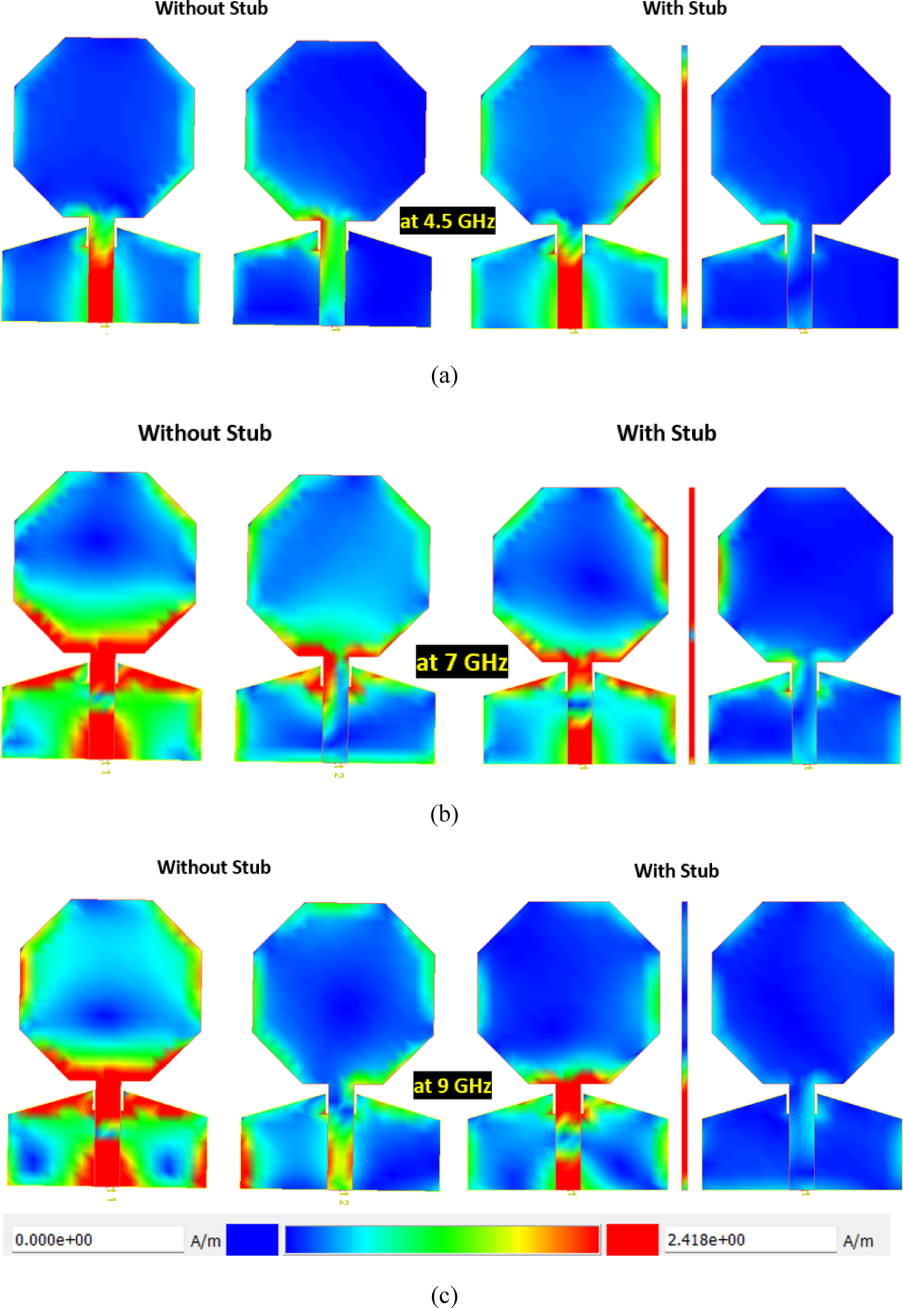
Surface current distribution of proposed antenna with and without current limiting stub (**a**) 4.5 GHz, (**b**) 7 GHz, (**c**) 9 GHz.

Figure 6 presents the surface current distribution of the proposed antenna at three different frequencies (4.5 GHz, 7 GHz, and 9 GHz), comparing configurations with and without the current-limiting stub. The left column shows the current distribution without the stub, while the right column depicts the distribution with the stub included. At 4.5 GHz (Fig. 6a), significant current coupling is observed between the ports in the absence of the stub, particularly along the feedline and radiating elements. The introduction of the stub mitigates this issue, redirecting and suppressing unwanted current flow. At 7 GHz (Fig. 6b), the current distribution without the stub reveals strong coupling and higher intensity near the shared sections of the structure, which is effectively minimized with the stub, demonstrating its ability to isolate the ports effectively. At 9 GHz (Fig. 6c), the scenario without the stub continues to exhibit substantial mutual coupling, while the stub effectively confines the current to the active port, reducing interference. The color scale further highlights the efficiency of the stub in limiting current propagation, ensuring enhanced isolation and improved performance across all analyzed frequencies. This analysis reinforces the stub’s critical role in minimizing coupling for MIMO or multi-port antenna applications.

**Fig. 7:**
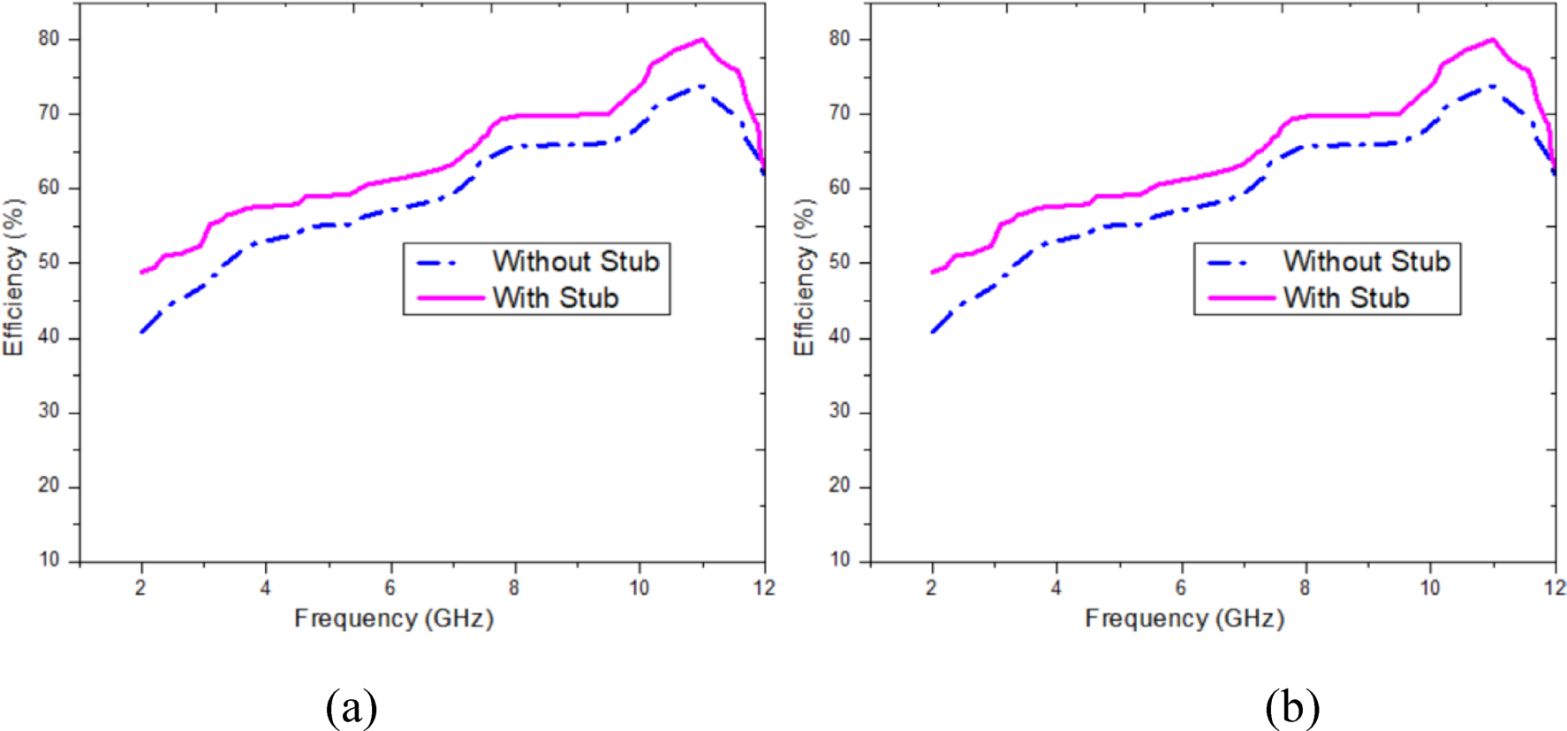
Effect of current limiting stub on (**a**) Gain, (**b**) Efficiency.

The figure demonstrates the effect of a current-limiting stub on system performance metrics across the frequency range of 1 GHz to 12 GHz. In Fig. 7a, the Gain (in dB) is significantly improved when the stub is included, particularly at higher frequencies, showcasing a more pronounced performance advantage. In Fig. 7b, the Efficiency (in percentage) is similarly enhanced with the stub, especially in the mid- to high-frequency range, highlighting improved energy transfer. The plots collectively indicate that the inclusion of the current-limiting stub optimizes the system by enhancing both gain and efficiency across the operational frequency spectrum.

**Fig. 8:**
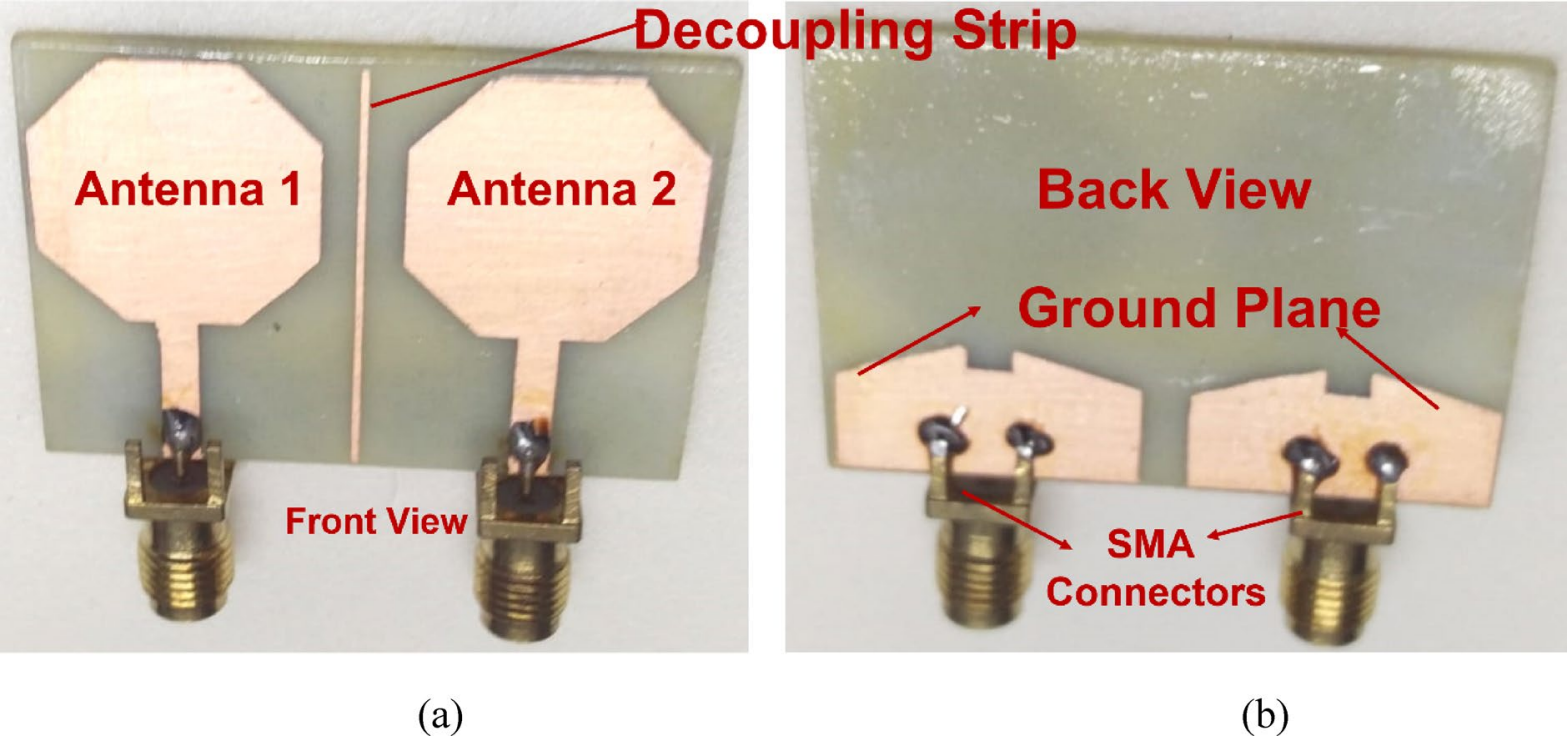
Fabricated images of the proposed antenna (**a**) Front view, (**b**) Back view.

**Fig. 9:**
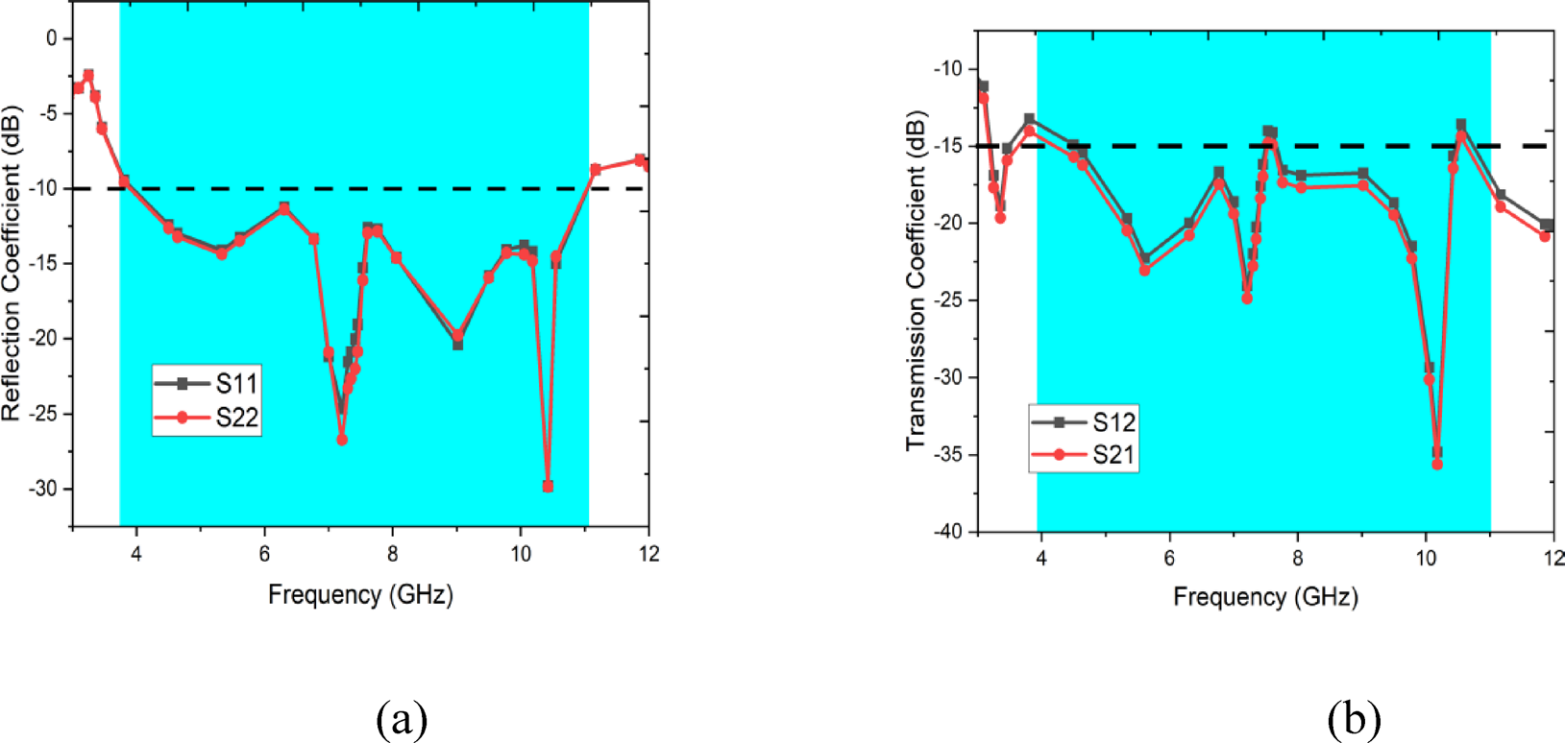
Measured (**a**) reflection coefficient, (**b**) transmission coefficient.

The fabricated design of the proposed two-port antenna is illustrated in Fig. 8a,b. Figure 9 illustrates measured reflection and transmission coefficients over a frequency range of 1 GHz to 12 GHz, providing insights into the performance of a two-port network. In Fig. 9a, the reflection coefficients, S11 and S22, are shown, indicating the amount of signal reflected back from each port. The plot reveals dips in the reflection coefficients at specific frequencies, implying good impedance matching at those points. In Fig. 9b, the transmission coefficients, S12 and S21, are presented, representing the signal transmission between the ports. Peaks in the transmission coefficients highlight frequencies where the network effectively transmits signals with minimal loss. These results collectively demonstrate the network’s frequency-dependent behavior, with optimal performance at frequencies corresponding to low reflection and high transmission.

**Fig. 10:**
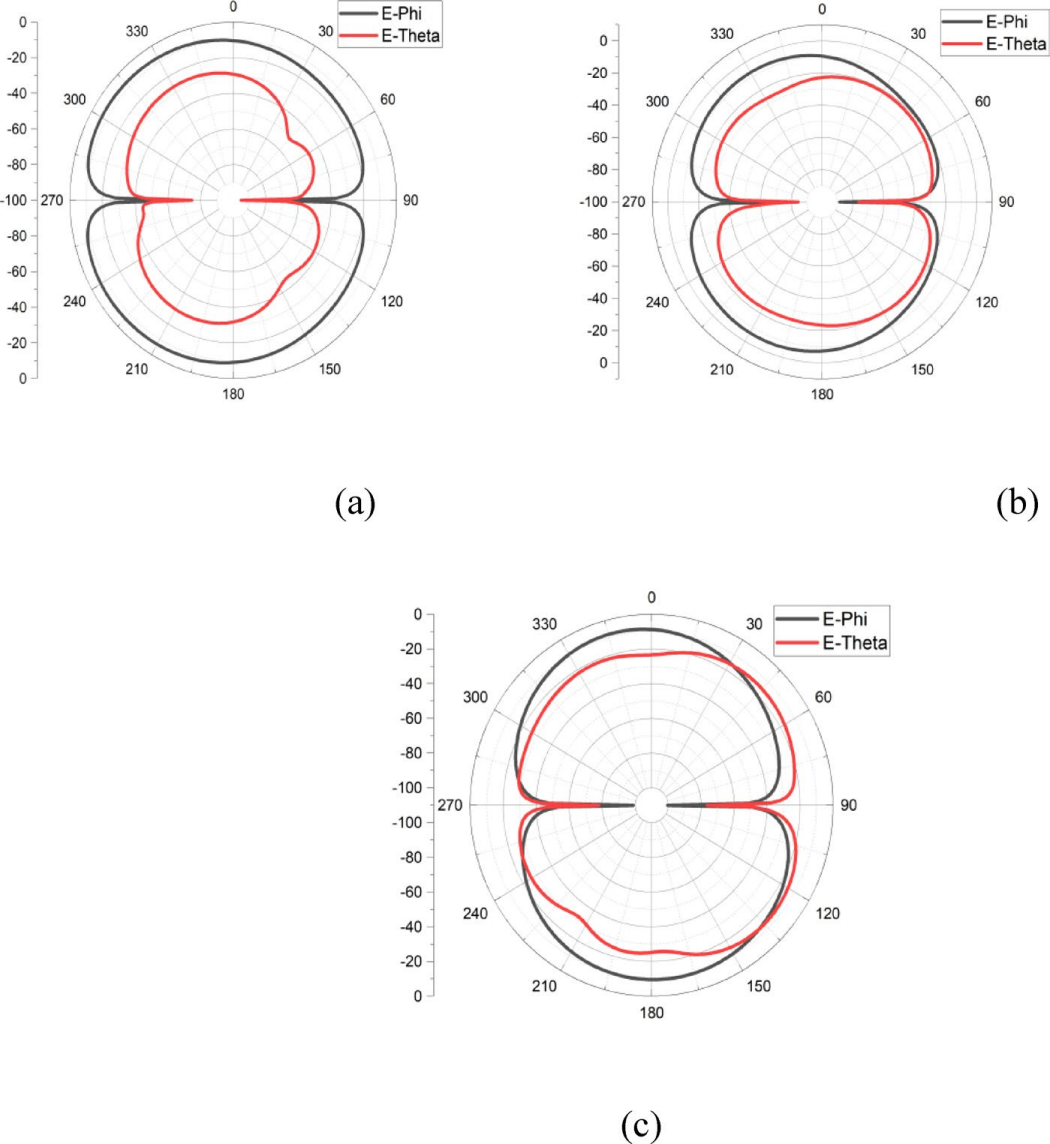
Measured 2D far field (E-Plane) pattern of the proposed antenna at (**a**) 4.5 GHz, (**b**) 7 GHz, (**c**) 9 GHz.

**Fig. 11:**
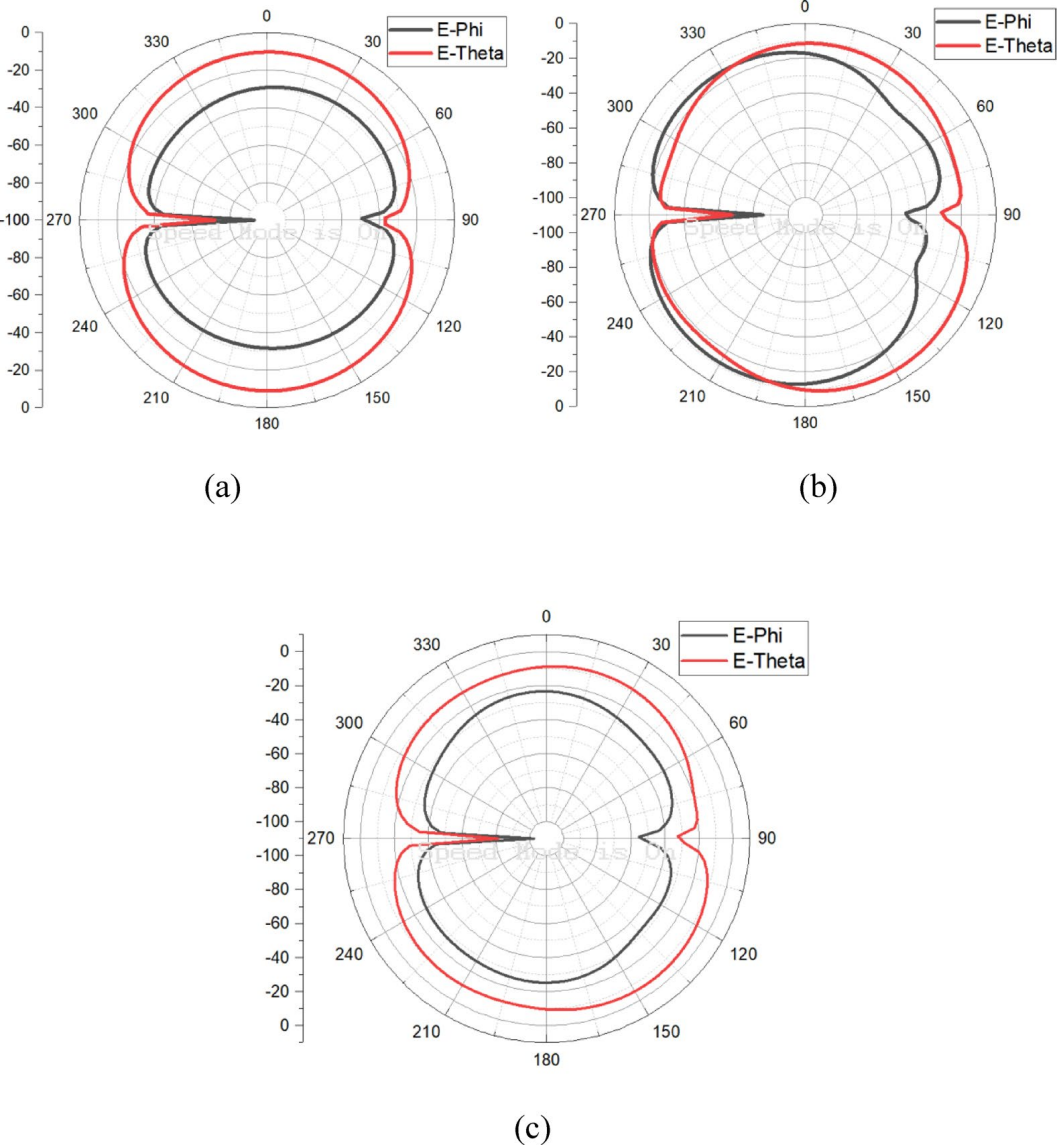
Measured 2D far field (H-Plane) pattern of the proposed antenna at (**a**) 4.5 GHz, (**b**) 7 GHz, (**c**) 9 GHz.

**Fig. 12:**
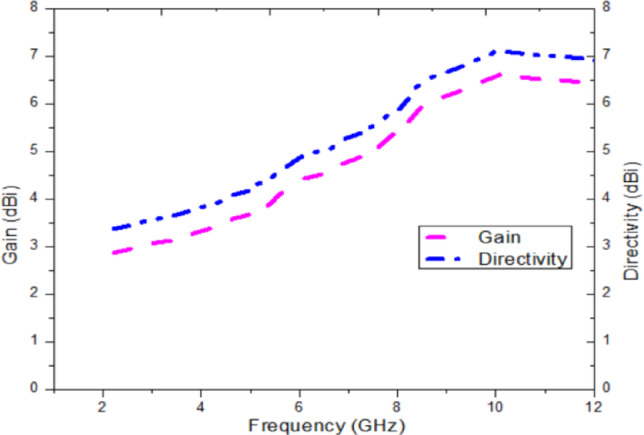
Measured gain and directivity of the proposed antenna.

The radiation patterns of the E-plane and H-plane for the proposed antenna at 4.5 GHz, 7 GHz, and 9 GHz are presented in Figs. 10a–c and 11a–c, respectively. Across all frequencies, the antenna exhibits distinct patterns for ϕ = 0° and ϕ = 90°, with cross-polarization suppression exceeding 20 dB. The measured gain and directivity are displayed in Fig. 12, with the antenna achieving a peak gain of 6.5 dBi and a directivity of up to 7 dBi within the operating frequency range. These characteristics ensure excellent performance for broadband applications.

## MIMO performance

**Fig. 13:**
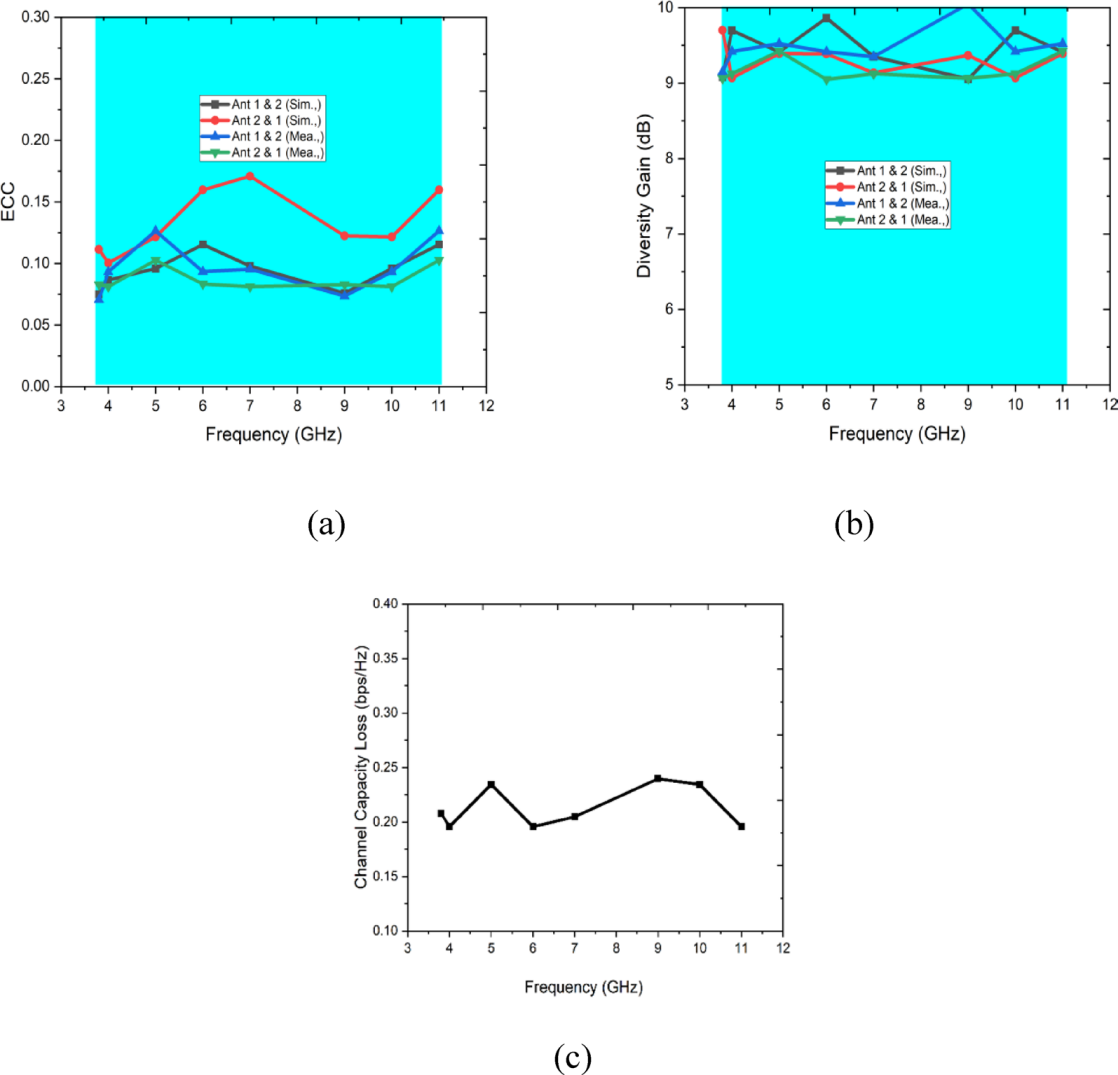
MIMO parameters (**a**) ECC, (**b**) Diversity Gain, (**c**) Channel Capacity Loss.

The figure illustrates key MIMO (Multiple-Input Multiple-Output) performance metrics across the frequency range of 3 GHz to 12 GHz, providing insights into the system’s behavior. In Fig. 13a, the Envelope Correlation Coefficient (ECC) between antennas is shown, which remains below 0.3 across the frequency range. This low ECC indicates good isolation and diversity performance between antenna elements. In Fig. 13b, the Diversity Gain (in dB) is displayed, which is near the optimal value of 10 dB across most frequencies, further confirming excellent diversity performance. Figure 13c presents the Channel Capacity Loss (in bps/Hz), which remains relatively low, suggesting minimal degradation in the system’s channel capacity. These results collectively demonstrate that the MIMO system achieves efficient spatial multiplexing and diversity, maintaining strong isolation, high diversity gain, and low channel capacity loss.

**Fig. 14:**
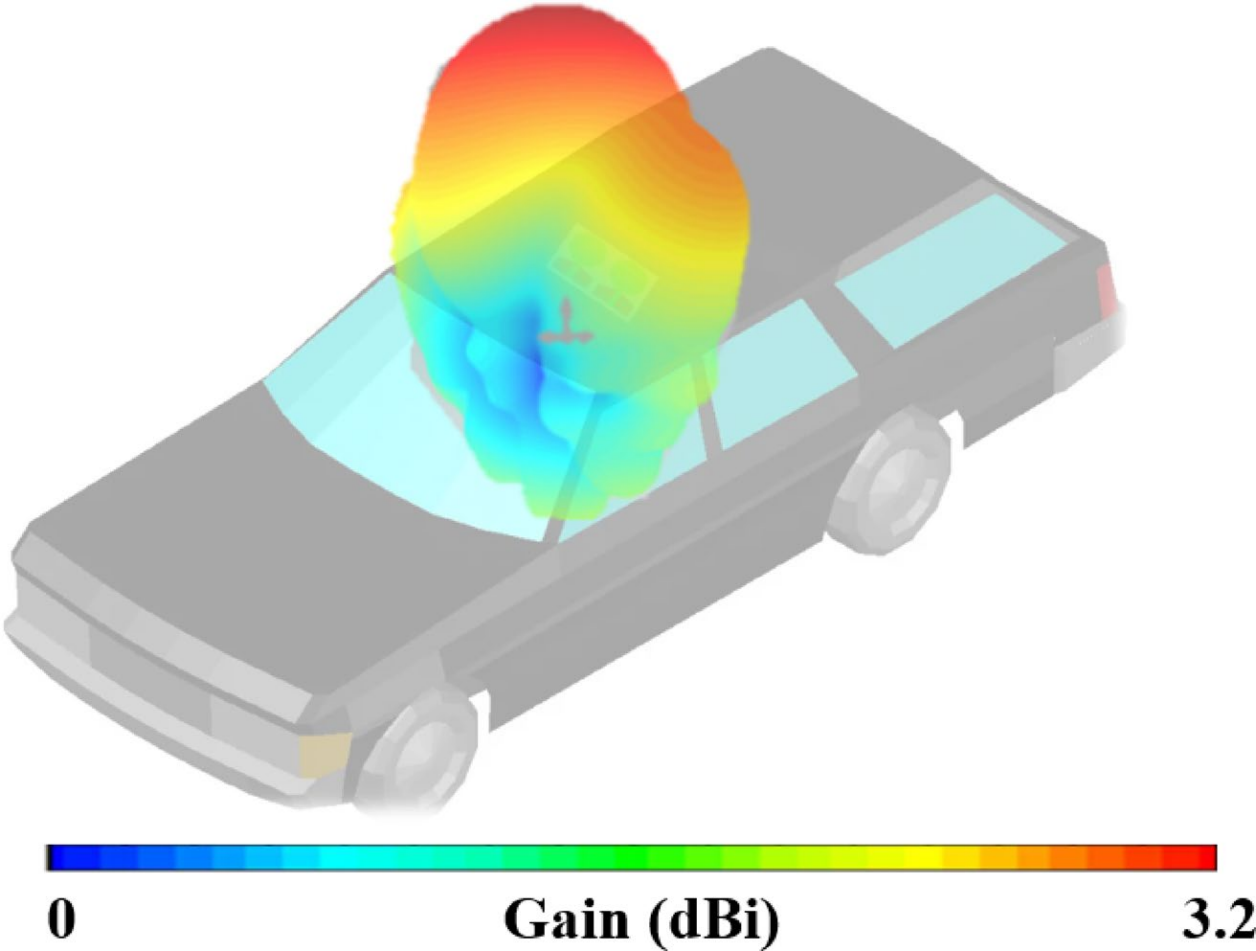
Placement analysis of proposed antenna at 5.9 GHz.

Figure 14 illustrates the placement analysis of the proposed antenna mounted on a car model at 5.9 GHz. It was generated using Microwave CST Studio 2021. The 3D gain distribution overlay demonstrates the radiation characteristics of the antenna in a real-world scenario. The colour scale, ranging from blue (0 dBi) to red (3.2 dBi), highlights the directional gain performance across the spatial domain. It is observed that the antenna exhibits a strong upward and slightly forward-directed gain pattern, with maximum radiation centered above the roof where the antenna is placed. This directional behaviour is crucial for vehicular communication applications such as DSRC (Dedicated Short Range Communications) and C-V2X (Cellular Vehicle-to-Everything), ensuring effective signal propagation toward roadside units and nearby vehicles. Moreover, the relatively symmetric pattern suggests that the antenna maintains good omnidirectional coverage in the horizontal plane, which is desirable for dynamic vehicular environments.

The proposed antenna design outperforms other works in the literature in Table 2 by achieving an exceptionally compact size of 16.2 × 25.6 mm^2^, the smallest among the compared designs, making it ideal for space-constrained applications. Despite the minimal inter-element separation of 3 mm, the design maintains strong performance, showcasing excellent isolation and diversity characteristics. Operating over a wide frequency range of 3.7 to 11 GHz, the antenna provides sufficient bandwidth for various wireless applications. It achieves a high peak gain of 6.5 dBi, surpassing most referenced designs, and maintains a low ECC of < 0.2, ensuring superior isolation and diversity performance. Furthermore, the antenna offers a high diversity gain of 9.8 dB and a competitive channel capacity loss (CCL) of 0.23 bps/Hz, balancing performance metrics effectively. These features highlight the proposed design’s ability to deliver high efficiency and robust performance in a compact and optimized configuration, surpassing many existing designs.

**Table 2:** Literature comparison of the proposed antenna.

Ref.No	Antenna size of the antenna (mm^2^)	Separation among radiating elements (mm)	Spectrum rangr (GHz)	Isolation (dB)	Peak gain (dBi)	ECC	DG (dB)	CCL (bps/Hz)
^14^	75.19 × 75.19	33	3.1–17.3	− 15.9	5	< 0.1	NR	NR
^17^	48 × 34	8	3.51–10.08	− 23	2.91	NR	9.81	< 0.29
^18^	80 × 80	NR	2.1–20	− 25	5.8	< 0.02	9.9	< 0.4
^19^	40 × 40	NR	1.8–3.94	− 25	2.36	< 0.4	9.71	0.13
^20^	40 × 40	4.25	3–13.5	− 15	3.5	< 0.012	9.95	0.22
^21^	74 × 74	NR	3.37–27.71	− 34	7.68	< 0.039	NR	NR
^22^	56 × 68	6	3.89–17.09	− 15	5.87	< 0.02	NR	< 0.5
^23^	30 × 30	9.75	3.1–12	− 17	6.2	< 0.001	9.9	0.1
Proposed	16.2 × 25.6	3	3.7 to 11	− 15	6.5	< 0.2	9.8	0.23

## Conclusion

A compact antenna system with close spacing and exceptional isolation was introduced, specifically designed for broadband usage across frequencies from 3.7 to 11 GHz. The system comprised two octagonal radiators, each powered by a 50 Ω microstrip line, and employed a modified ground structure to achieve a wideband frequency range. Despite a 3 mm separation between the ports, the system maintained over 15 dB of isolation. To enhance inter-port isolation, a stub measuring 25.6875 × 0.5 mm^2^ was incorporated between the radiating sections. The system’s performance, evaluated through fabrication and testing, demonstrated excellent alignment between simulation and experimental data. Key metrics, including envelope correlation coefficient (ECC), diversity gain (DG), and channel capacity loss (CCL), confirmed the MIMO efficiency. With a peak gain of 6.5 dBi and excellent resonance, the antenna proved highly suitable for broadband wireless systems.

## Data Availability

The data used to support the findings of this study are included in the article. Data will be provided on request from Rajesh Kumar D, Email: sdrk87@gmail.com and R Dhananjeyan, Email: dhananjeyanr.ece@valliammai.co.in
